# Supplementary figures and images for: A nap to recap or how reward regulates hippocampal-prefrontal memory networks during daytime sleep in humans
Source: eLife. 2015 Oct 16;4:e07903. doi: 10.7554/eLife.07903 (PMC4721959; doi:10.7554/eLife.07903)

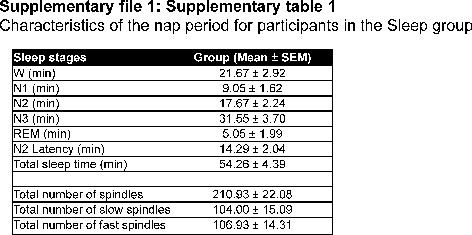

Supplement: Supplementary file 1. — DOI: http://dx.doi.org/10.7554/eLife.07903.015 [file elife-07903-supp1.jpg]

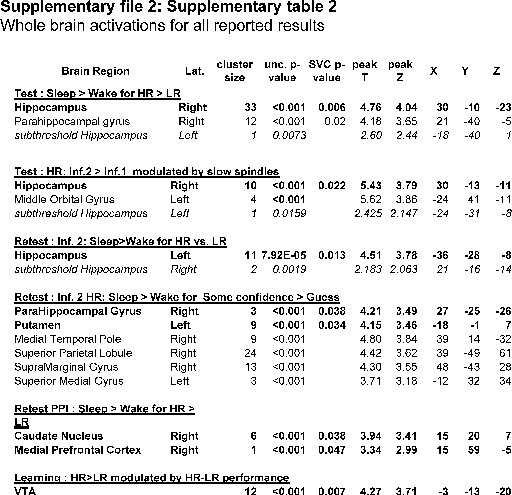

Supplement: Supplementary file 2. — DOI: http://dx.doi.org/10.7554/eLife.07903.016 [file elife-07903-supp2.jpg]
